# Exploring augmented grasping capabilities in a multi-synergistic soft bionic hand

**DOI:** 10.1186/s12984-020-00741-y

**Published:** 2020-08-25

**Authors:** Cristina Piazza, Ann M. Simon, Kristi L. Turner, Laura A. Miller, Manuel G. Catalano, Antonio Bicchi, Levi J. Hargrove

**Affiliations:** 1 Department of Physical Medicine and Rehabilitation, Northwestern University, Chicago, 60611 IL USA; 2 The Regenstein Foundation Center for Bionic Medicine, Shirley Ryan AbilityLab, Chicago, 60611 IL USA; 3 Istituto Italiano di Tecnologia, Genoa, 16163 Italy; 4 Centro “E. Piaggio” and Dipartimento di Ingegneria Informatica, University of Pisa, Pisa, 56122 Italy; 5 Department of Biomedical Engineering, Northwestern University, Evanston, IL USA

**Keywords:** Bionic hand, Adaptive synergies, Soft robotics, Myoelectric control

## Abstract

**Background:**

State-of-the-art bionic hands incorporate hi-tech devices which try to overcome limitations of conventional single grip systems. Unfortunately, their complexity often limits mechanical robustness and intuitive prosthesis control. Recently, the translation of neuroscientific theories (i.e. postural synergies) in software and hardware architecture of artificial devices is opening new approaches for the design and control of upper-limb prostheses.

**Methods:**

Following these emerging principles, previous research on the SoftHand Pro, which embeds one physical synergy, showed promising results in terms of intuitiveness, robustness, and grasping performance. To explore these principles also in hands with augmented capabilities, this paper describes the SoftHand 2 Pro, a second generation of the device with 19 degrees-of-freedom and a second synergistic layer. After a description of the proposed device, the work explores a continuous switching control method based on a myoelectric pattern recognition classifier.

**Results:**

The combined system was validated using standardized assessments with able-bodied and, for the first time, amputee subjects. Results show an average improvement of more than 30% of fine grasp capabilities and about 10% of hand function compared with the first generation SoftHand Pro.

**Conclusions:**

Encouraging results suggest how this approach could be a viable way towards the design of more natural, reliable, and intuitive dexterous hands.

## Introduction

Capturing the richness and complexity of the sensory-motor functions of the human hand in a prosthetic device remains one of the challenge in modern science and engineering [1]. State-of-the-art commercial prostheses include sophisticated poly-articular hands, designed to match the appearance and function of human hands through the ingenious combinations of multiple motors and sensors [2]. The classical approach to manage their advanced dexterity consists of using a pair of surface electromyographic (sEMG) sensors to control one degree of freedom (DoF) at a time and switch between several motion patterns through different input strategies [3]. Muscles trigger sequences, such as co-contractions [4], are among the most used techniques in commercial devices. Alternative solutions include control through mobile apps, the use of short-range proximity sensors, or, the combination between sEMG sensors and inertial measurements units [5]. Unfortunately these hi-tech devices are often overcome by simple non-anthropomorphic hook-like systems, usually preferred for their reliability, robustness and simplicity of control [6, 7].

To address current limitations, clinical and engineering research is investigating new strategies to improve the level of acceptability of these advanced devices, e.g. minimizing the cognitive effort or increasing their robustness. From a clinical point of view, two successful and innovative approaches are those based on Targeted Muscle Reinnervation (TMR) [8, 9] and intramuscular EMG [10], via wireless transceivers [11] or an osseointegrated implant [12]. All of these techniques considerably increase users’ capabilities to selectively activate several muscles in a more natural fashion, and consequently to control multiple DoFs. From an engineering point of view, significant improvements over conventional methods are given by the introduction of simultaneous and proportional myoelectric control using linear regression techniques [13, 14], which create a continuous map between EMG signals and the intended movements or pattern recognition algorithms, mostly based on the information of muscles groups. The latter are exploited following several techniques [15–19], but a widely used approach is based on a classifier [20–22]. Over the past two decades, pattern recognition technology has shown promising results in providing more intuitive control of myoelectric prosthesis, and has been incorporated into commercial devices.^1^^,^^2^ However, while pattern recognition guarantees higher robustness within each identified class, it introduces some restrictions in terms of flexibility (i.e., commercial systems require the full re-opening of the device in order to switch between different grips). Recently a deeper knowledge of the human sensory-motor architecture and the adoption of soft robotic technologies are contributing to the development of a new generation of more efficient bionic limbs. The neuroscientific concept of postural synergies [23] is one of the most explored approaches and consists of the introduction of coordinated finger movements, which takes inspiration from human motor control principles. This approach demonstrated its effectiveness in several aspects of the development of artificial hands, including kinematic studies [24], control architectures of multi degrees of actuation (DoA) hands [25–27] and also mechatronic design [28]. Looking at the signal processing and control field, a promising approach consists on postural control algorithms based on principal component analysis [29], which map the principal component coordinates into hand joint angles. This method allows to continuously control different grips, increasing the control intuitiveness of multi-DoA hands [26]. In response to the need for increased device reliability and robustness, in the last decade, there are evident trends toward a strong simplification of the system in terms of the number of actuators and sensors adopted [30], i.e. through the introduction of underactuation and hand synchronized motion. Still exhibiting an anthropomorphic design, these novel devices are capable of performing a useful subset of the functions of human hands, with a consequent reduction in terms of control complexity and an increase of device reliability [31–33]. Taking advantage of the studies on human postural synergies [34], the SoftHand Pro (SH-P) [28] is anthropomorphically designed with 19 DoFs, soft roll-articular joints and a single actuation unit. Although its structure is almost comparable to the one of the human hand, the physical synergistic architecture allows to control the SH-P with only two sEMG sensors. This design strategy introduces the possibility to perform complex physical interaction tasks, higher robustness and intuitiveness, while still demonstrating good skills in terms of grasp capabilities. Such encouraging results suggest to extend these principles towards hands with enhanced skills, but still capable to maintain an intrinsic simple and robust architecture and a reasonably natural control method. To deal with this dexterity-complexity trade-off, we proposed the Pisa/IIT SoftHand 2 (SH2) [35], which tries to embed the advantages of the synergy-based postural control directly in the hand mechatronic design. The SH2 still has 19 DoFs but, using two actuators to reproduce approximately the first two synergies of the human hand, shows a higher level of dexterity.

**Fig. 1:**
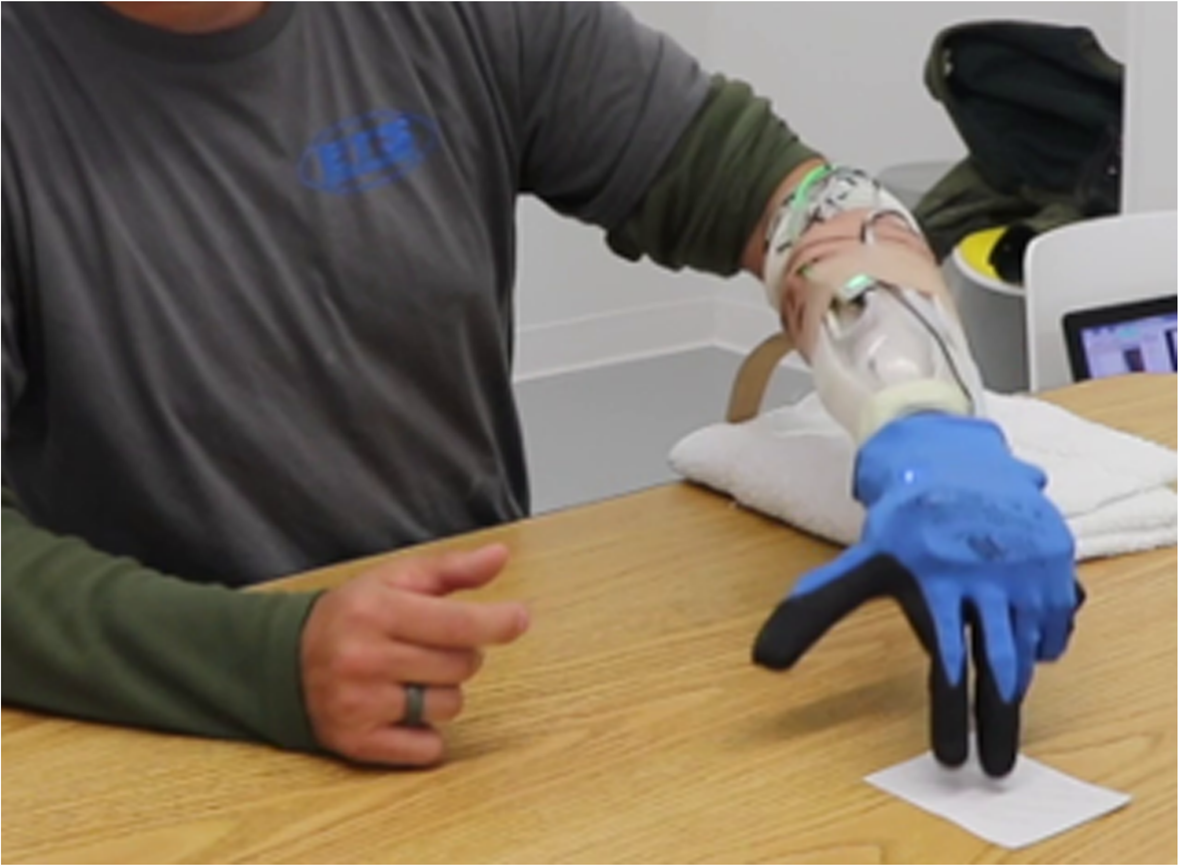
An amputee subject moving a card with the SoftHand 2 Pro. The index point grasp is used to execute the task

In this work, we introduce the SoftHand 2 Pro (SH2-P), the prosthetic release, characterized by a light-weight design and suitable to be connected with a prosthetic socket and multi-channel sEMG system (see Fig. 1). To extensively investigate the role and efficacy with real prosthetic users, this study presents the SH2-P controlled with a pattern recognition classifier and an ad-hoc multi-channel sEMG system explicitly designed for clinical use. Similar to the approach successfully tested with postural control algorithms, this work implements a continuous switching method to move between different grip patterns able to introduce more flexibility to conventional pattern recognition classifier. The user is able to seamlessly shift the hand between grasp positions without moving along pre-defined open and close positions for each grip. The resulting system can be controlled in a simultaneous, proportional and continuous way, avoiding the drawback of conventional approaches, which requires stopping in an initial position (usually hand-open) in order to switch between different grips (or patterns). A preliminary validation of this approach with only 3 able-bodied subjects was presented in a previous work [36]. This study includes for the first time also amputee subjects which tested the device using standardized functional assessments (Box and Blocks Test and Jebsen-Taylor Hand Function Test) and a subjective survey. Results show improved fine grasp capabilities and performance compared with the SoftHand Pro and suggest how the approach can be a viable way towards the design of more natural and intuitive dexterous hands. The rest of the paper is organized as follows: “Materials and methods” presents the prosthetic prototype, the control algorithm and a description about participants, experimental setup and protocol. The results on both able-bodied and amputee subjects are presented in “Results” and compared with the SH-P and other research prototypes and control methods from literature. “Discussion” discusses the insights gained from these results and the limitations of the study. Finally, “Conclusion” sections draws the conclusions of our work.

## Materials and methods

### SoftHand 2 Pro

**Fig. 2:**
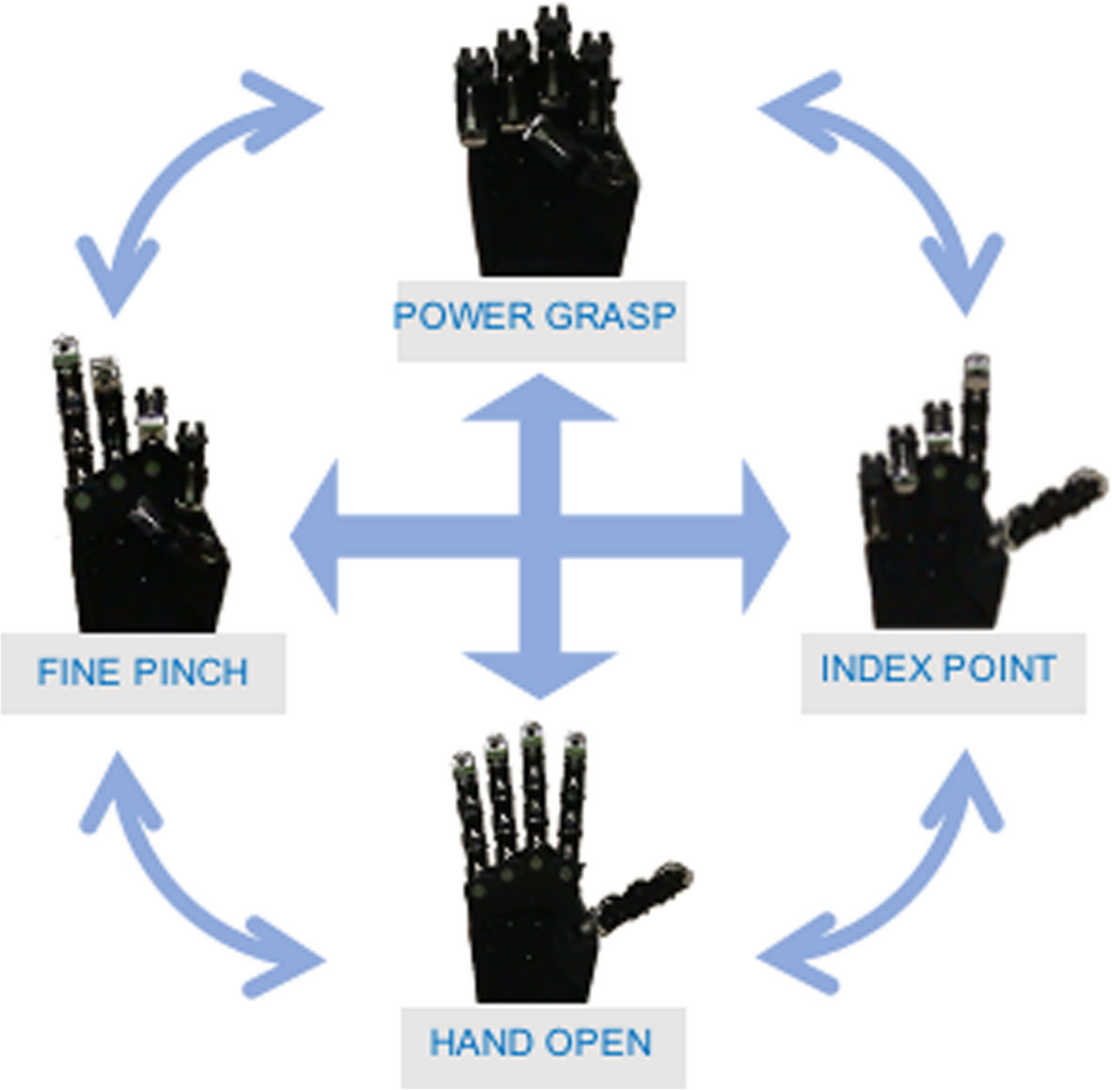
Representation of the defined gestures of the SoftHand 2 Pro and the possible path generated by the simultaneous activation of the 2 DoA. Note that the movement activated along the vertical direction reproduces the same movement of the SoftHand Pro

The SoftHand 2 is a soft articulated anthropomorphic robotic hand with 19 DoFs, which can perform sophisticated tasks with a higher level of dexterity but using only two motors (see Fig. 2). Indeed, through the physical introduction of hand coordinated movements, it is possible to considerably reduce the architectural complexity in terms of actuators and sensors, and consequently increase the device robustness and reliability. Preliminary investigations on the robotic prototype have been done in a previous work [37], where authors explored the possibility to use specific signal maps relying only on two EMG signals. The SH2-P is capable of spanning continuous movements in the synergy bi-dimensional space, through the simultaneous and coordinated activation of the two actuators. To fully exploit such feature in a previous work [38], authors investigated the possibility to control the hand in a proportional and simultaneous way relying on the use of an off-the-shelf low cost multi-channel system (a MYO Arm Band). Although only preliminary (one healthy subject testing the robotic release of the hand), results reported in a previous work [38] show the hand capabilities while performing activities of daily living, and possible benefits that can be encountered from using continuous and proportional control strategies.

Since the promising results achieved by previous works, a new version called SoftHand 2 Pro (SH2-P) was designed to test its extended functionalities in the prosthetic field. The new prototype uses two MAXON DCX 16s 12V motors coupled with 83:1 gearboxes, to guarantee the same performances of the SH-P [28], but with an effective reduction of the hand size and, consequently, of weight.

**Fig. 3:**
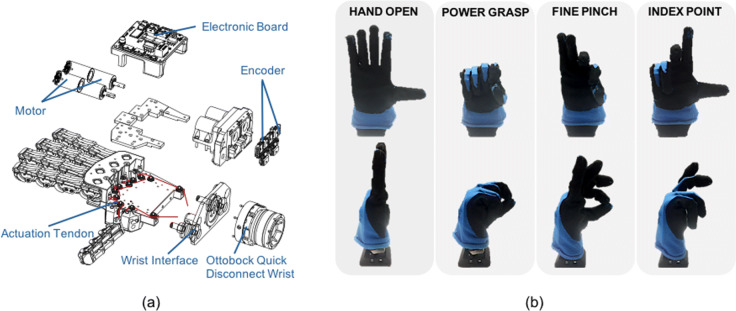
SoftHand 2 Pro design. Panel **a** shows the exploded view of the SoftHand 2 Pro, with the main components highlighted. SoftHand 2 Pro presents a self-contained design, where the two motors, electronic board and sensors are integrated in the dorsal side of the prototype. The prototype is equipped with an Ottobock quick disconnect prosthetic wrist. Panel **b** shows the frontal (top) and lateral (bottom) view of all the possible SoftHand 2 closures: Hand Open (first column), Full Finger Closure (second column), Pre-shape Fine Pinch (third column) and Pre-shape Index Point (fourth column). Full Finger Closure reproduces the same movement of the SoftHand Pro

Table 1 shows a comparison between the robotic and the prosthetic releases. As presented in the exploded view of Fig. 3a, the novel hand design is self-contained, and integrates the actuation system, sensors and a custom electronic board [39] directly in the palm. The device is also equipped with a passive pronation-supination Ottobock quick disconnect prosthetic wrist. Thanks to the use of a single actuation tendon that moves from the palm base through all the fingers and exploiting the effect of the device transmission friction, the SH2-P is capable to span continuously its motions in the synergy space, reaching specific points where it is possible to identify definite grasping postures (showed in Fig. 3b):

**Table 1:** Hand Characteristics

Specifics	SoftHand 2	SoftHand 2 Pro
Dimension	25 x 15 x 6.7 mm	21 x 15 x 5 mm
Weight	712 gr	491 gr
Motor	Maxon DCX 22s	Maxon DCX 16s
Reduction Rate	83:1	83:1
Voltage	12V	12V

i *Hand Open*, the rest position with the fingers fully open;
ii *Power Grasp*, a configuration with all the fingers fully closed, useful to grasp big or heavy objects;
iii *Fine Pinch*, a configuration where the thumb goes in opposition with the index, more indicated for precision grasps of small objects,
iv *Index Point*, a configuration where the index is pointing and other fingers are closed, useful to press buttons or keys.

**Fig. 4:**
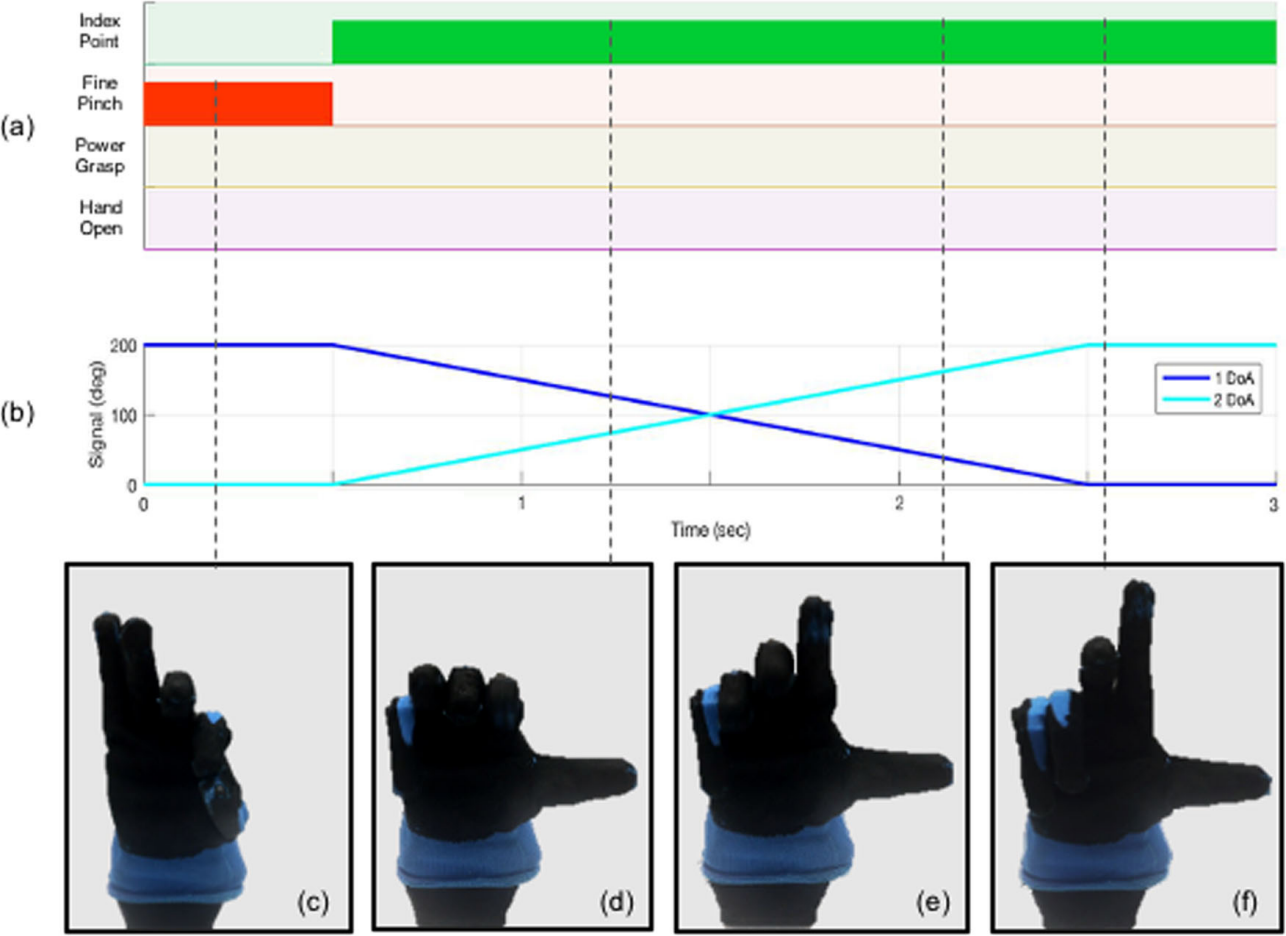
An example of the system driving the hand control from fine pinch to index point. Panel **a** shows the pattern recognition classifier and the reference motor position of both actuators is presented in **b**. Once the classifier detects the index point class, the system commands the actuators of the artificial hand towards the new gesture. The control input is proportional to the muscle activation and commands the two motors in opposition, to move the hand from fine pinch to index point. It is also possible to stop in intermediate configurations

The guidelines of its design take inspiration from the neuroscience-based notion of soft synergies [23] and their translation into augmented adaptive synergies [35]. This approach allows users to exploit frictional effects to achieve advanced motions, with the aim to increase the performance of the device without over-raising the complexity of the whole system. The first degree of actuation implements a coordinated closure of all fingers (see Fig. 2, vertical direction) correspondent to the first synergy of grasp in humans [23], which is a very fundamental ingredient of human hand control [40, 41]. The second degree of actuation implements relative motion between the fingers, as shown in Fig. 2 (horizontal direction). The opening and closing movements of left fingers with respect to right ones (and vice versa) is found in the second and third postural synergies of grasp [23], the second manipulation synergy [42], in the second synergy of haptic exploration [40], and in the third synergy of environmental constrain exploitation in [41]. We believe that the implementation of a similar motion could be a valid instrument to embed a higher level of dexterous capabilities. A more detailed discussion can be found in a previous work [35]. Furthermore, in-hand manipulation skills can be obtained moving the hand from fine pinch to index point, as showed in the hand movements of Fig. 4. Please refer also to the video attachment for more details. In past research, several valuable approaches exploit the use of a synergistic method to control multi-DOFs hands [24, 25, 29]. However, in the SH2-P such synergistic layers are physically implemented in the mechanical architecture of the hand, and not reconstructed from specific control algorithms that coordinate multiple motors acting on individual fingers. This leads to a considerable reduction in terms of actuators unit and sensors required, with the aim to find a proper trade-off between hand dexterity and device robustness.

### Continuous switching control method

**Fig. 5:**
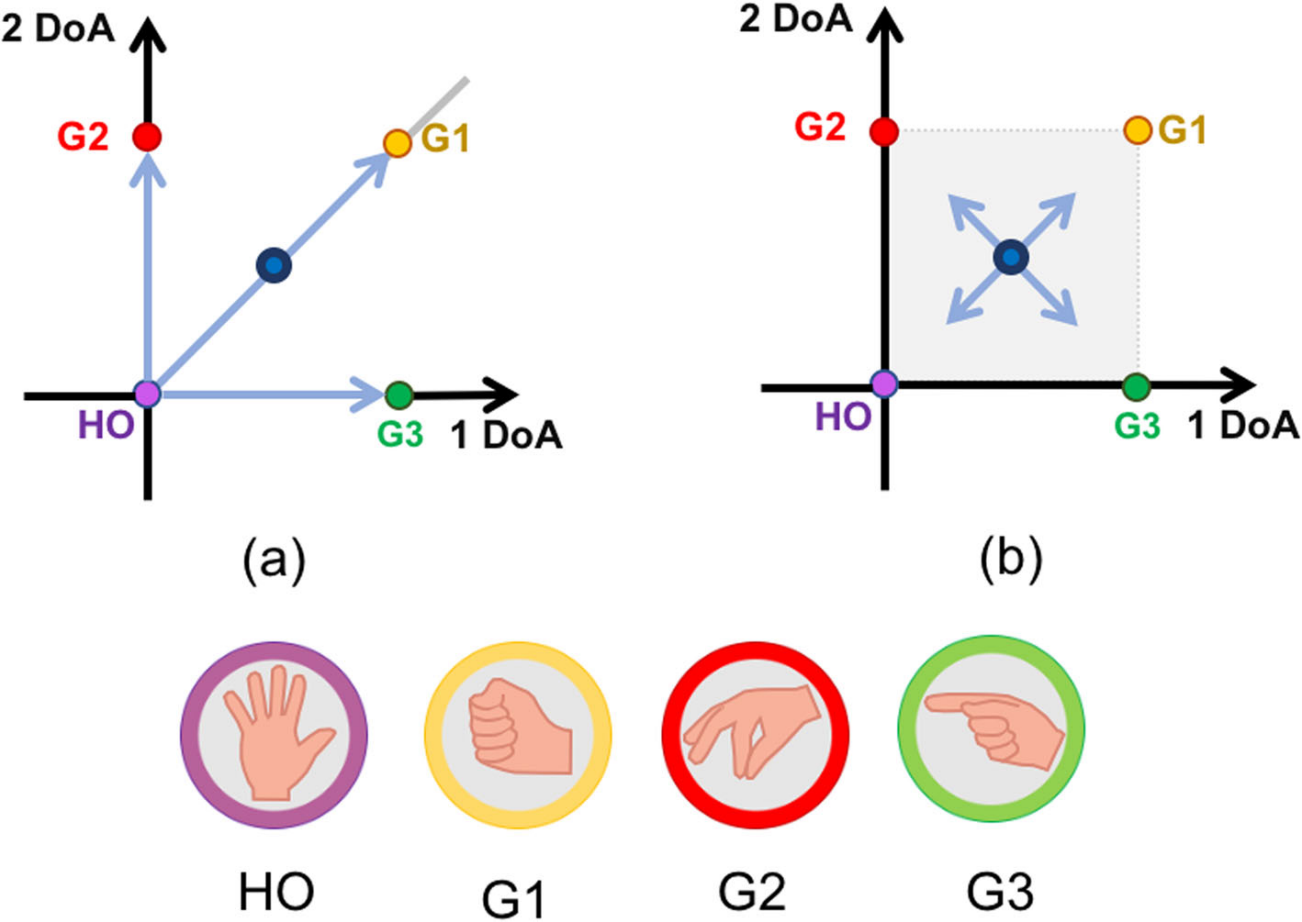
Schematic representation of a standard myoelectric pattern recognition classifier **a** and the proposed continuous switching method **b**. The blue dot refers to the current hand position, while the other coloured dots represent four different hand final positions, described as HO, G1, G2, G3 in this example. The classical approach require to reach the hand open state (HO) to enable the switch between different gestures (i.e. G1, G2, G3,..), while the proposed method allows to move continuously from one gesture to another

A widely used approach to control advanced hand dexterity consists of using pattern recognition techniques based on a classifier. Pattern recognition control methods are based on the assumption that the residual limb is rich in information about the intended movements and these data can be clustered in groups and used to identify different motions. The extracted information is fed into a classifier, which is trained to recognize selected hand postures. Once the system is in the initial position and a defined grasp pattern is identified, the device is commanded to move and reproduce the associated posture. Although possible in principle, one of the main drawbacks of such approach, when used in combination with off-the-shelf devices, still remains the incapability to have proportional and continuous control of all the joints of a multi-digit hand. This is mainly due to the mechatronic architecture of commercial anthropomorphic hands and to the limited possibilities of integration (usually off-the-shelf systems do not allow access to low-level features). Consider a fully actuated prosthetic hand whose mechanics allow up to three different gestures (i.e. called G1, G2, G3) and hand open (HO) as an initial position. Using an opportune number of sEMG sensors and a pattern recognition classifier, each combination of muscles contractions of the user is identified with the appropriate class of motion and drives control of different hand postures. However, the use of a classification method in combination with commercial prosthetic hands, requires to reach the initial state to enable the switch between different gestures, limiting intuitive control. As schematically shown in Fig. 5a, the actual position of the hand (represented by the blue point) can be proportionally controlled only along the path between the initial hand open position (HO) and the recognized gesture (i.e. G1, G2, G3). The method proposed in this work, which we call the *continuous switching control*, implements a classifier that allows to move continuously from one gesture to another, as represented in Fig. 5b, exploiting the main features of the SH2-P. In this case, when the activation pattern is recognized, the hand prototype is able to immediately reach the new state, overcoming the requirement to stop in an intermediate position. The change of state is determined by the classifier. If the pattern recognition algorithm detects a new motion class (with an activation value over a settled threshold), a motor control command is immediately sent to the prosthesis controller, which maps the features extracted from the EMG signals into motor reference positions. The motor positions are acquired using magnetic sensors, as showed in Fig. 3a.

Figure 4 presents an example, where the system drives the hand control from fine pinch (c) to index point (f). Once the algorithm detects a minimum level of contraction necessary to initiate the index point, the system directly commands the actuators of the artificial hand towards the latest recognized gesture. The control input, which is proportional to the muscle activation, moves the two motors in opposite directions and actuates all joints at the same time through a unique tendon, resulting in a re-opening of the thumb and index finger and a closure of the other three fingers.

The use of two different synergistic directions with the characteristic hand mechanics allow to obtain combined movements which cover the whole space represented in grey in Fig. 5b, without requiring the introduction of additional classes in the pattern recognition algorithm. The same method can potentially be applied to a fully actuated robotic hand through a proper control algorithm, while in the SH2-P is done by exploiting the specific architecture.

### Participants

Five able–bodied subjects took part in the preliminary experimental evaluation (3 males and 2 females, 4 right-hand and 1 left-hand dominant) with no impairments and ages between 21 and 29 years old. The system was also tested with 3 trans-radial amputee subjects (see Table 2), two of which had previously undergone TMR surgery. All the subjects were unilateral amputees, two of which are myoelectric users and one is a body-powered user. Subjects were not blinded to the pattern recognition control method but they never tested the SH2-P and the continuous switching method. The following study was approved by the Northwestern University Institutional Review Board and all participants gave their informed consent.

**Table 2:** Amputee subjects demographics

	Subj. 1	Subj. 2	Subj. 3
Age	31	55	26
Gender	M	M	M
Side of amputation	Left	Right	Right
Time since amp. (yrs)	4	39	8
Time since TMR (yrs)	1.5	NA	2
Home Device	Myoel.	Myoel.	Body Pow.
PR Exp. Level	High	Low	Low

### Experimental setup

**Fig. 6:**
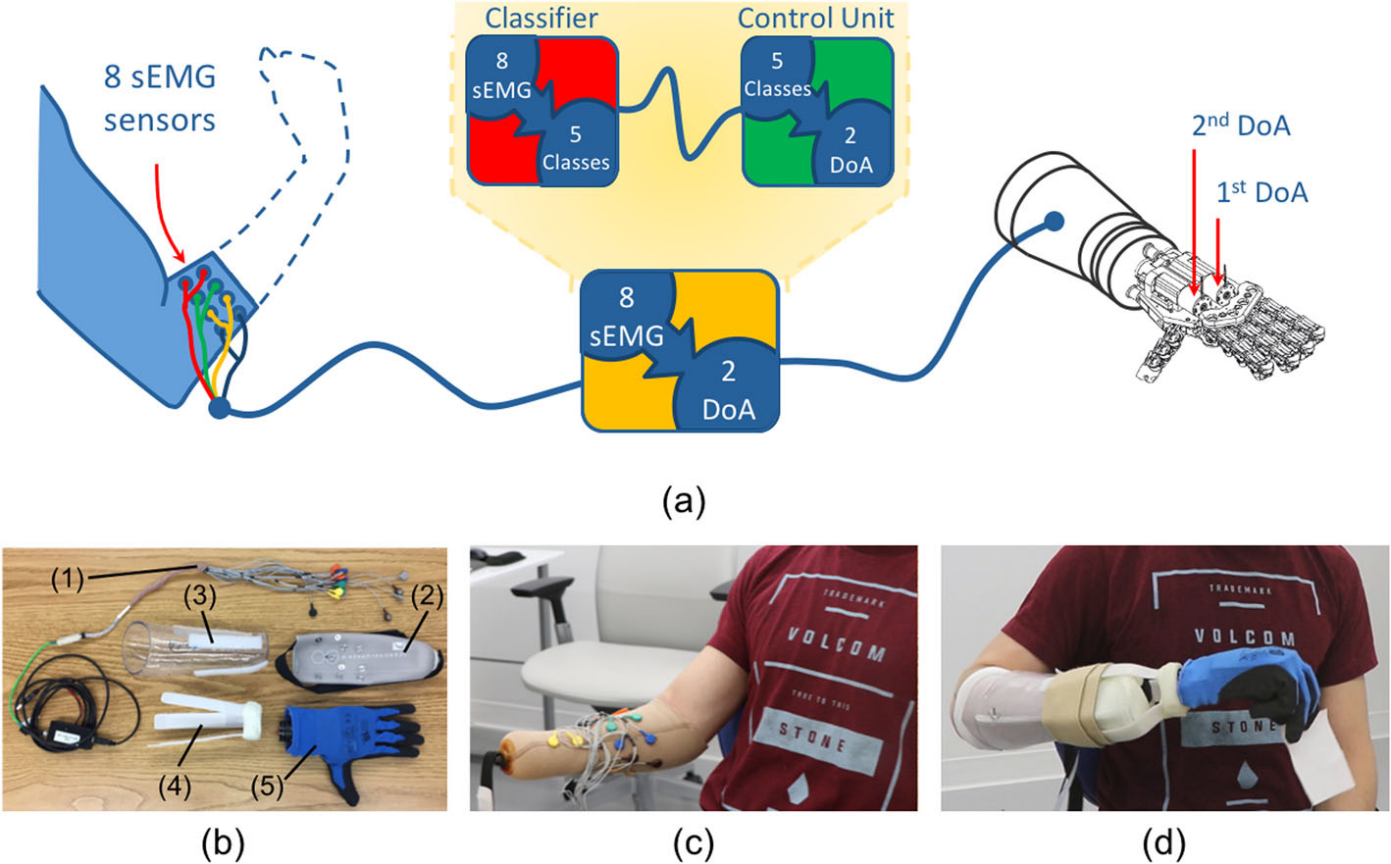
Panel **a** shows a schematic of the proposed control architecture. Myoelectric signals collected from 8 sEMG sensors were processed using linear discriminant analysis (LDA) classifiers and grouped in 5 classes: no movement, hand open, power grasp, fine pinch and index point. These classes were used to control the define grips of the SoftHand 2 Pro through the simultaneous activation of the 2 DoA. Panel **b**-**d** show the experimental setup used by amputee subjects: **b** shows hardware components including 8 sEMG sensors (1), the customized liner (2) and socket (3), a support interface compatible with the Ottobock quick disconnect wrist (4) and the SoftHand 2 Pro prototype (5); **c** presents an example of surface electrodes distribution on an amputee subject; **d** shows the system worn by a user during the experimental session

The control architecture used in this work is schematically presented in Fig. 6a. The myoelectric signals are collected from the forearm of the user through a group of 8 sEMG sensors. Each subject wore eight equally-spaced pairs of stainless steel dome electrodes and one reference electrode (Motion Control Inc.), with inter-electrode spacings of approximately 2.5 cm. Signals were sampled at 1 kHz using a Texas Instruments ADS1299 bio-instrumentation chip. A multi-class linear discriminant analysis (LDA) classifier [43] was trained for five classes, using the Control Algorithms for Prosthetics System (CAPS), developed by the University of New Brunswick (Fredericton, New Brunswick, Canada) and Rehabilitation Institute of Chicago (Chicago, Illinois), now known as Shirley Ryan AbilityLab. The selected classes correspond to the defined gestures of the SH2-P: no movement, hand open, power grasp, fine pinch and index point (represented in Fig. 3b). To guarantee good classification accuracies [16, 44], four time-domain features (mean relative value, waveform vertical length, zero crossings and slope sign changes) were extracted from each channel over 250 ms windows with 100 ms overlap (selected based on previous work [45]). Data were band-pass filtered between 30-350Hz.

Data were collected and applied to train a linear discriminant analysis classifier and features from the test set were used to test the classifier’s accuracy (averaged over all the movements to calculate the overall classification accuracy). A Python v3.6 code was implemented to linearly translate each class in a corresponding control command of the 2 DoA of the SH2-P in a spatially and temporally continuous way. A linear function was implemented to command the hand movement from one position to another. When the hand is in an intermediate position between two extreme postures of the space domain (e.g. G2 and G3 of Fig. 5) the fingers reach an intermediate position between the two extreme configurations. Such kind of behaviour is highlighted in Fig. 4 where the fingers move from one state to another in a continuous way, but can also assume intermediate positions, as e.g. in snapshots (d) and (e). The continuous gesture recognition allows switching between different postures without the need to reach an initial position and simultaneously commands both actuation units. In the SH2- P this approach is translated in a combination of the sum and/or difference of the position of the two motors. Moreover, thanks to the mechanical design of the SH2-P, a continuous switching control between pinch grasp and index point allows in-hand manipulation, without the introduction of an additional class.

#### Able-bodied subjects

A wearable mechanical interface was used to connect the SH2-P to the human operator forearm. The experimental setup is similar to the one used to test the SH-P in previous work [28]. It consisted of a plastic shell with the prosthetic hand attached on the bottom part. The user forearm was locked to the mechanical interface through two velcro strips. The hand could be shifted along the interface and was placed approximatively 3 cm distal to the operator’s hand. Each subject wore a cuff embedded with eight surface EMG sensors. The cuff was positioned with the reference electrode distal to the elbow on the posterior side of the arm and adjusted to provide proper coverage around the circumference of the forearm.

#### Amputee subjects

As shown in Fig. 6b, one reference electrode and eight pairs of stainless steel dome electrodes (1) were embedded in a prosthetic gel liner (2). An example is showed in Fig. 6c. The subject then donned a customized socket (3) that, with an interface compatible with an Otto-bock quick disconnect wrist (4), creating an easy interface with the hand prototype (5). The whole experimental setup used was fitted by a trained prosthetist. Figure 6d shows the experimental socket worn by one subject during the session. This setup is similar to the ones adopted in a previous work [28], except for the number of electrodes used.

### Experimental protocol

To evaluate the system performance, an experimental protocol was designed following the three steps presented in this subsection. The first part consisted of the calibration of the system, followed by a training session and then by the experimental session (conducted with an open clinical trial). The same protocol was used with able-bodied and amputee subjects.

#### System calibration

The subject was seated in front of a screen and was asked to mimic the movement prompted in the CAPS software. The pre-programmed sequence of motions was showed using a graphical user interface and was repeated three times for each of the five movements collected. Each movement was collected for 3 seconds, followed by three seconds rest. To avoid fatigue, subjects were allowed 2 minutes of rest between trials. To increase the robustness of the classifier [46] and limit the limb position effect [26], subjects wore the bypass or the prosthetic device during the data acquisition and they were asked to move their arm in different positions while they were performing each gesture. In this way, it was possible to consider signals changing due to different limb positions and to limit eventual classification errors. After all the data were collected, the pattern recognition classifier was automatically trained from the software. The average classifier accuracy was 95% for able-bodied subjects and 93.5% for amputee subjects.

#### Training session

**Fig. 7:**
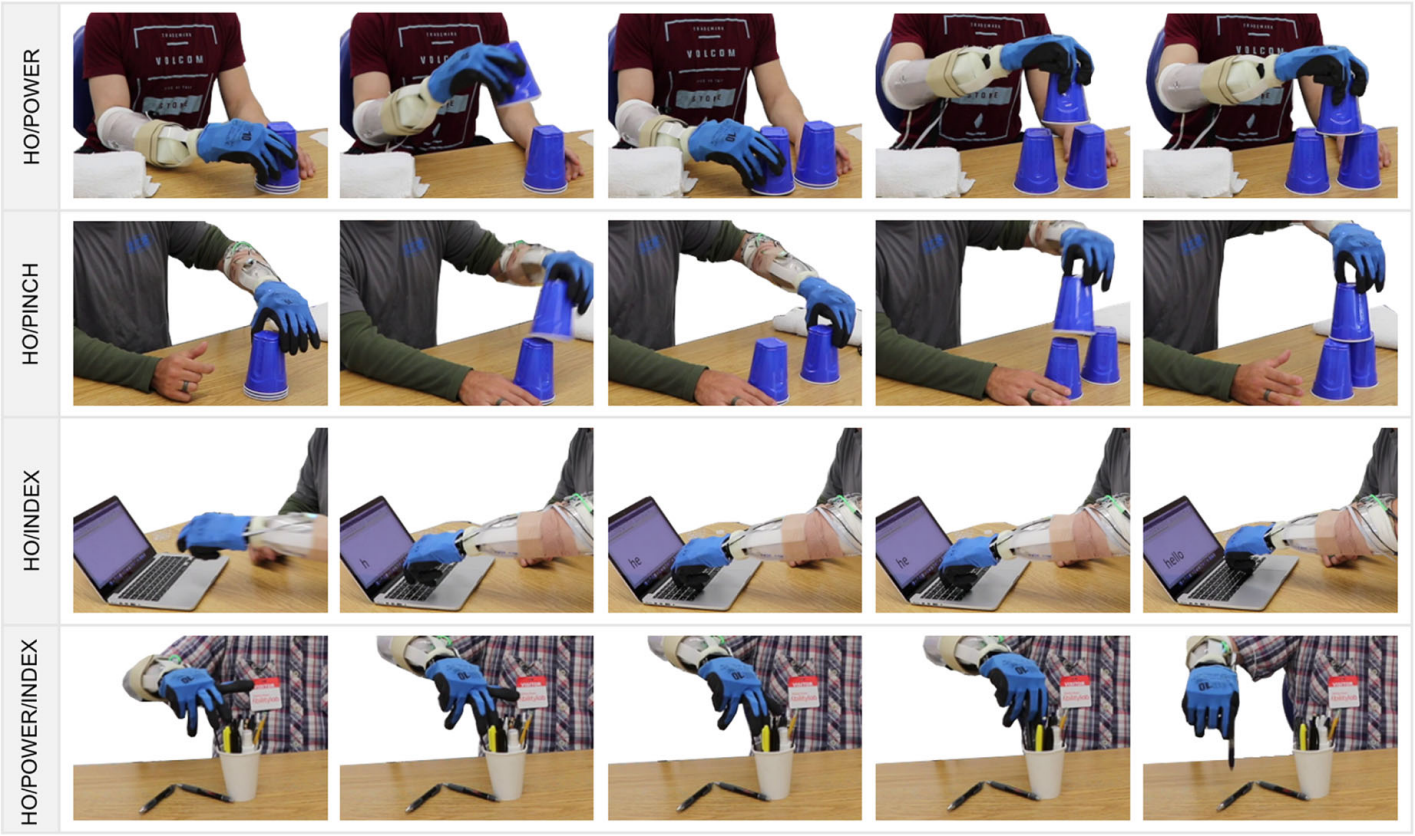
Examples of training activities. In the first row the amputee subject was building a cup pyramid using only power grasp, while in the sequence showed in the second row a participant is using only the pinch grip. In the pictures presented in the third row the pre-shaping index point is used to write a word on a computer, to increase precision and visibility while performing the task. Finally, in the last row the subject is switching between index point and power grasp to picking a pen from a penholder

Since all the subjects were naive to the SH2-P, a training session was useful to explore the hand functionalities and how to exploit the hand compliance and adaptability, features that are not common in off-the-shelf devices. On average, the training session was about 1 hour and 30 minutes. During this session, the subjects were guided to learn each gesture and the combination of them. The first part of the training was devoted to learning and becoming familiar with the device capabilities. The tasks included in this training session, designed with the help of an occupational therapist, were selected to optimize hand closure, and to improve the precision manual dexterity and control stability. The subjects were able to experience 1 DoA per time, performing a simple generic task and an activity. The first hand motions practiced were power grasp/hand open and then fine pinch/hand open. In both cases, the subjects were asked to grasp a sequence of objects with different shapes and weight (for example, a bottle, a toothbrush, a credit card, etc), and build/demolish a cup pyramid of 2 cups base. An example of this task is showed in the first and second row of Fig. 7. To practice the index point movement, the subjects were asked to drag small objects (i.e. wooden cube, coins) from one side to another of the table and try to write a word using a computer keyboard (see the third row of Fig. 7). The following step focused on the combination of three motions (power grasp/hand open/index point), to get familiar with the control method. This session was useful also to set the activation threshold values for each class and each subject. At this point, more advanced tasks were proposed to the subjects, such as turning a page of paper or turning cards upside down. In the example shown in the last row of Fig. 7, the user experienced the advantages of the continuous switching strategy to grasp a pen from a penholder. The subject used the index point to move the pen to a more convenient grasping position and then switched directly to a full closure to grasp it. The training was considered completed when the user was able to reach a certain level of control capabilities, in order to avoid that their level of experience with pattern recognition could influence the results.

#### Experimental session

**Fig. 8:**
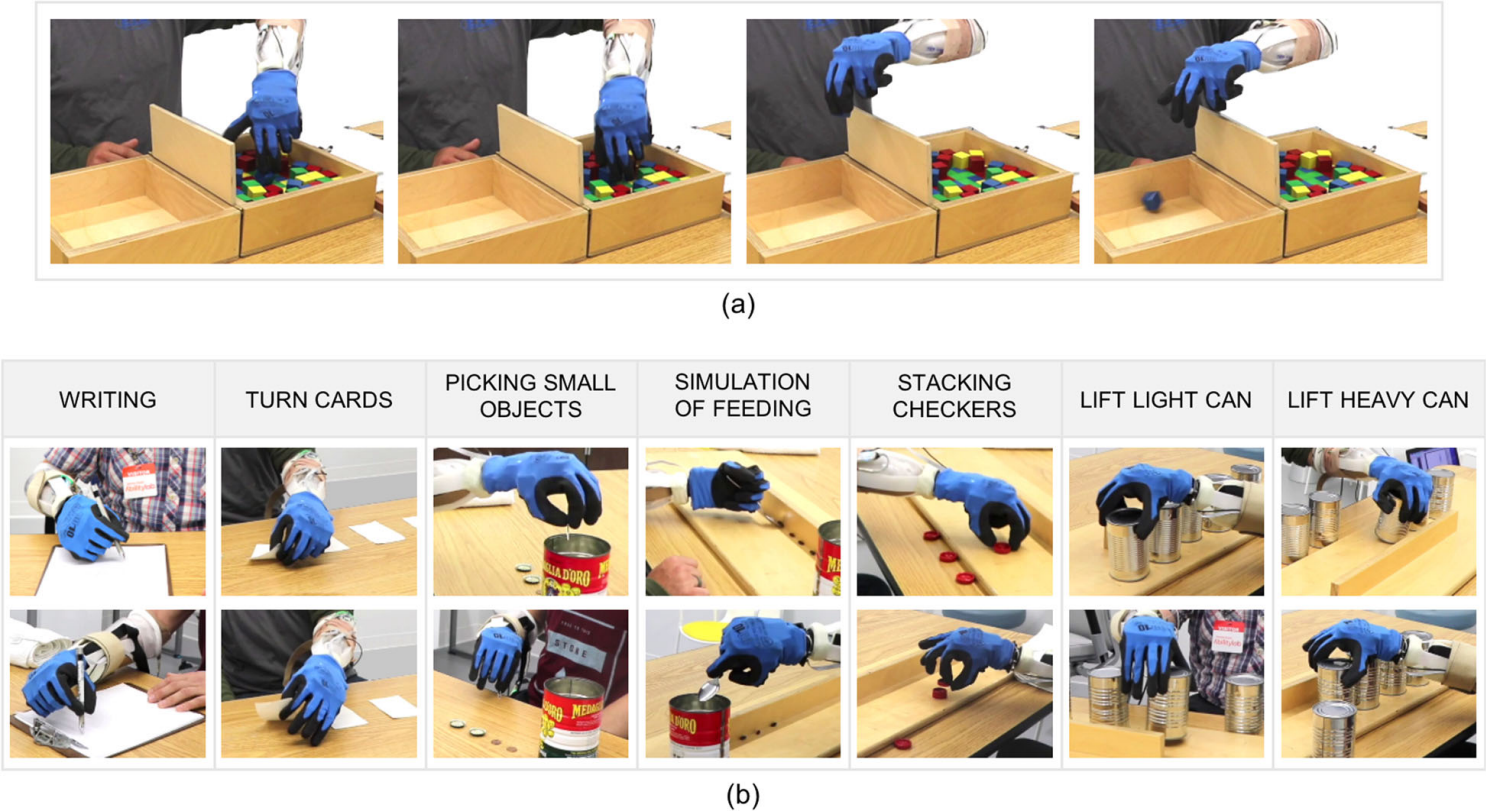
Amputee subjects testing the SoftHand 2 Pro performing the Box and Blocks **a** and the Jebsen-Taylor Test **b**. The photo sequence in **a** shows an example of BBT performed by an amputee subject. The participant was using the continuous switching between classes to perform the test. The pictures in **b** show different grasp strategies to complete the same task, possible thanks to features as the adaptability and dexterity of the SoftHand 2 Pro
- I was able to grasp objects/perform the task very easily
- It was easy to reach the chosen closure
- The switch between different closures was intuitive
- I’m not tired at all

After training, the subjects performed two standard assessments. During each test, the subjects were free to choose the preferred gesture (or the combination of gestures) to complete the task. Since the SH2-P was never tested with amputee subjects, the first assessment used for this validation was the Box and Blocks test (BBT) [47], a test that is widely used to study the usability of prosthetic devices [48]. Subjects are instructed to move as many wooden cube blocks as possible from one box to the adjacent one in sixty seconds. The blocks are placed in a random orientation on the first box, which adds variability to each trial. During the test, the users were allowed to move more than one block at a time, but it was counted as one. To obtain a more consistent dataset, the subjects performed three repetitions of sixty seconds, interspersed with sixty seconds break. A sequence of the experiment is shown in Fig. 8a. The hand functions for activities of daily living were evaluated through a second assessment, the Jebsen-Taylor Hand Function Test (JTT) [49]. This test consists on 7 sub-tasks: writing a 24-letter sentence, turning 5 cards, picking up small objects and placing it in a box, stacking 4 checkers, a simulation of feeding and moving first light and then heavy cans. A time-out was considered at 120 sec for each task. An overview of the JTT sub-tasks is presented in Fig. 8b. The maximum time to complete each sub-task is 120 seconds, and the score is given by the total time required to accomplish all the sub-tasks. At the end of the functional test, the subjects were asked to complete a questionnaire to evaluate the level of satisfaction with the device, the control and switching method, and the level of fatigue experienced during the experiments. A 5-points Likert-like scale was used for this evaluation, rating the level of agreement by a value from 1 to 5. The sentence were:

- The hand was easy to control

## Results

**Fig. 9:**
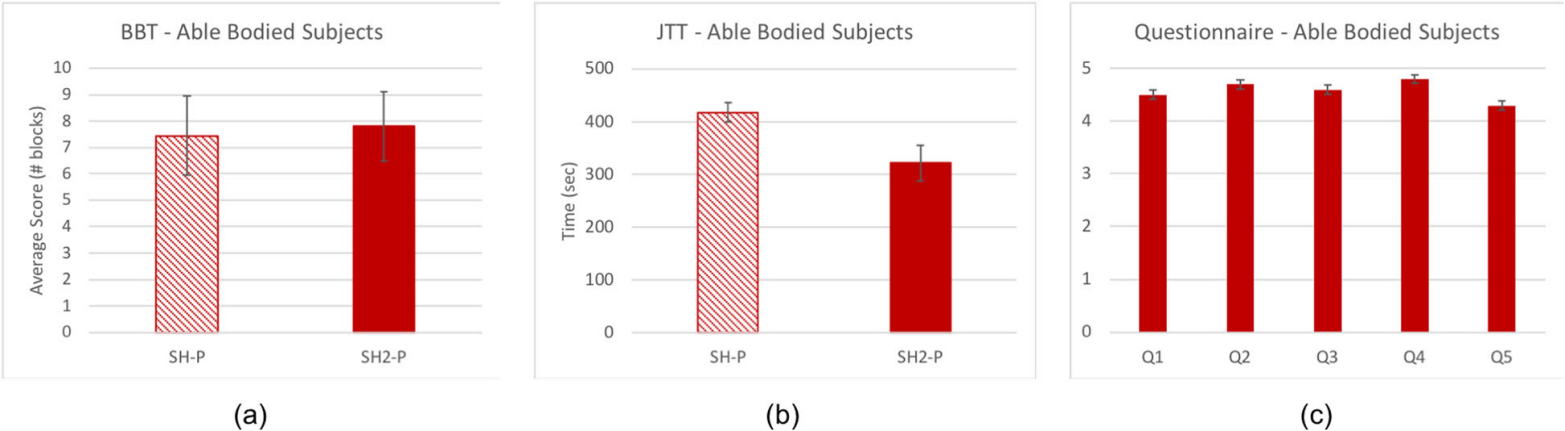
Average results with the standard error on able bodied subjects performing standard assessment: **a** BBT and **b** JTT. The performance of the SoftHand 2 Pro (in red) are compared with the SoftHand Pro (in striped red - data extracted from [28]). At the end of the protocol the subjects were also asked to complete a questionnaire. Evaluation of the questionnaire statements (min 1, max 5) are reported in **c**; (Q1) The hand was easy to control, (Q2) I was able to grasp objects/perform task very easily, (Q3) It was easy to reach the chosen closure, (Q4) The switch between different closures was intuitive, (Q5) I’m not tired at all

**Fig. 10:**
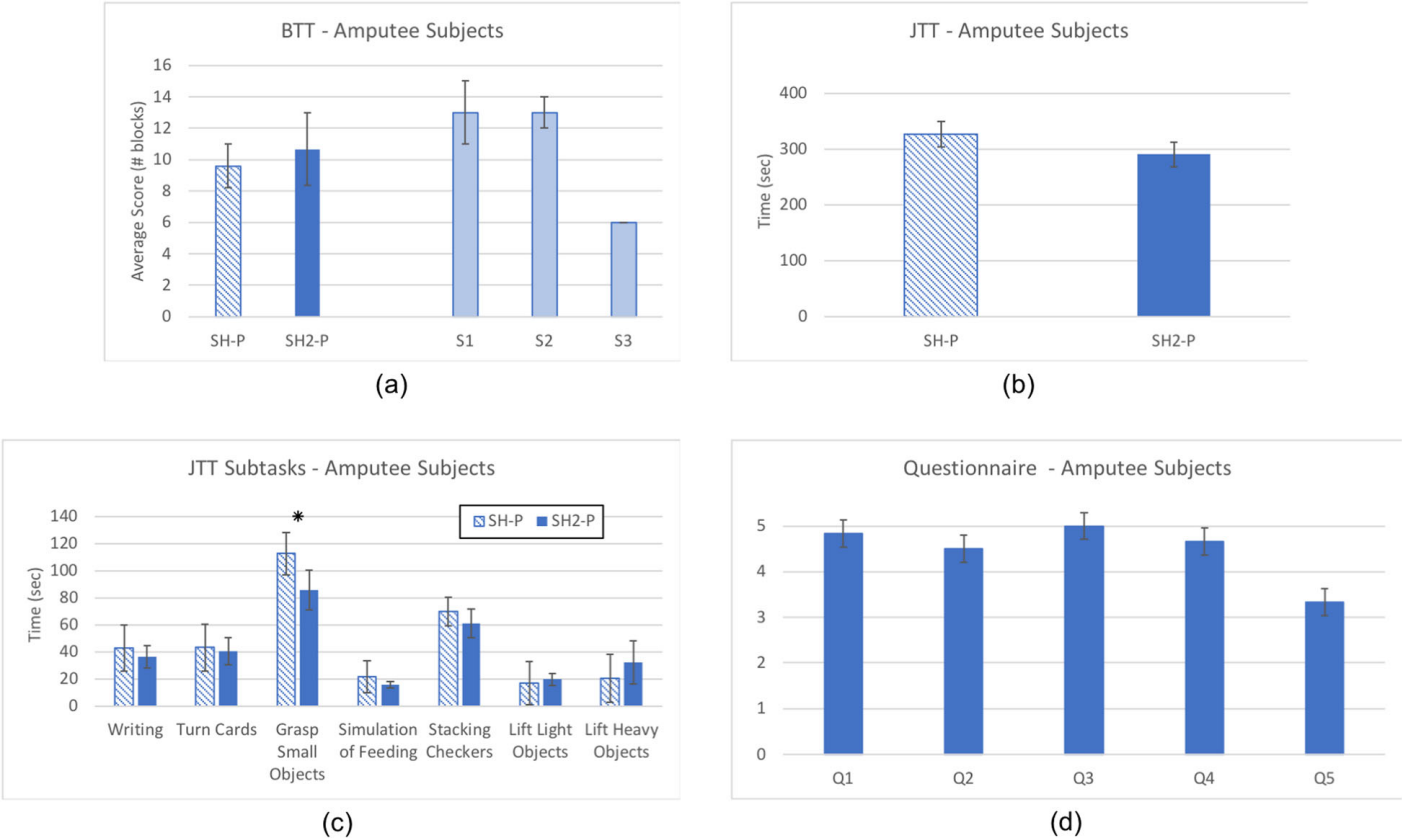
Average results with the standard error on amputee subjects performing standard assessment: **a** BBT and **b** JTT. The performance of the SoftHand 2 Pro (in blue) are compared with the SoftHand Pro (in striped blue - data extracted from [28]). An average of the score for each JTT subtask is showed in **c**. At the end of the protocol the subjects were also asked to complete a questionnaire. Evaluation of the questionnaire statements (min 1, max 5) are reported in **d**; (Q1) The hand was easy to control, (Q2) I was able to grasp objects/perform task very easily, (Q3) It was easy to reach the chosen closure, (Q4) The switch between different closures was intuitive, (Q5) I’m not tired at all. Statistically significant comparison (p <0.05) are denoted with a “*”

To evaluate the extended design features of the SH2-P, results are compared with the SH-P (using data extracted from a previous study [28] and the same standard assessments). Figure 9 presents results on able-bodied subjects, while Fig. 10 shows the outcome on amputee subjects. A two-sample t-test and a two-way analysis of variance with ANOVA is conducted to evaluate the differences between the two groups of subjects (with and without limb loss), the two hand devices (SH-P and SH2-P), and the interaction between them. Statistical significance for all tests was set at 0.05. Means are reported as *mean ± standard error.*

To analyze the performance of the SH2-P and the proposed control method with a more wide perspective, results are also compared with other studies from literature, that use the same standard assessments to evaluate research prototypes and control strategies. Table 3 presents the average results for able-bodied subjects and a comparison with the SH-P (using data extracted from [28]), the Delft Cylinder Hand (using data extracted from [50]) and the Ottobock Electric Hand (using data extracted from [45]). Table 4 shows the average results for amputee subject and a comparison with the SH-P (using data extracted from [28]), the Hosmer 5XA body-powered hook and the Motion Control Electric Device (using data extracted from [51]), a Switch-Controlled hand and the Ottobock 8E44 DMC Plus (using data extracted from [53]), the Michelangelo Hand controlled with Direct Control and Pattern Recognition Control (using data extracted from [52]).

**Table 3:** Average results for Able-Bodied Subjects using SH2-P and comparison with the SH-P (data from [28]), the Delft Cylinder Hand (data from [50]), the Ottobock Electric Hand (data from [45])

Terminal Device	Control	BBT	JTT	Quest
SH2-P	Myoelectric (8 sEMG sensors)	7. 8 ±1.3	321.2 ±33.7	4.58 ±0.5
SH-P [28]	Myoelectric (2 sEMG sensors)	7.4 ±1.5	417.5 ±17.9	NA
Delft Cylinder Hand [50]	Body-Powered Harness	17 ±6 / 26 ±8 ◇	NA	NA
Ottobock Electric Hand [45]	Myoelectric (2 sEMG sensors)	25 ±3.5 / 28 ±4 ◇◇	NA	NA

◇data from 1st/15th trial

◇◇data from slow/fast devices

**Table 4:** Average results for Amputee Subjects using SH2-P, and comparison with the SH-P (data from [28]), the Hosmer 5XA body-powered hook and the Motion Control Electric Device (data from [51]), the Switch-Controlled hand and the Ottobock 8E44 DMC Plus (data from [53]), the Michelangelo Hand controlled with Direct Control and Pattern Recognition Control (data from [52])

Terminal Device	Control Method	BBT	JTT
SH2-P	Myoelectric Cont. PR (8 sEMG sensors)	10.67 ±2.3	290.75 ±33.7
SH-P [28]	Myoelectric DC (2 sEMG sensors)	9.6 ±1.4	327.46 ±22.8
Hosmer 5XA Hook [51]	Body-Powered Harness	49	NA
Motion Control Electric Device [51]	Myoelectric DC (2 sEMG sensors)	20	NA
1 DoF Hand [53]	Switch-Controlled (humeral abduction)	6	621
Ottobock 8E44 DMC Plus [53]	Myoelectric DC (2 sEMG sensors)	2 / 8 ◇	769/567 ◇
Michelangelo Hand [52]	Myoelectric DC (2 sEMG sensors)	14.7 ±9/8.7 ±5.5 ◇◇	362 ±135.1/331 ±52.1 ◇◇
	Myoelectric PR (6 sEMG sensors)	5.7 ±1.2/11 ±6.1 ◇◇	346.3 ±77.7/332.3 ±69.3 ◇◇

◇Data before/after 8 weeks training with a single subject

◇◇Data before/after home trial

### Subject comparison using SoftHand 2 Pro

The average score of the BBT was *7.8 ±1.3* for able-bodied subjects and *10.67 ±2.3* for amputee subjects, both using the SH2-P. For the JTT, the total average score of the test was *321.2 ±33.7* for able-bodied subjects and *290.75 ±33.7* for amputee subjects. The average results for the amputee subjects are presented in Table 3, while the results of the three amputee subjects for each test are reported in Table 5. Two-sampled t-test indicated that there is not sufficient evidence to reject the null hypothesis that the result of BBT and JTT for able-bodied subjects and amputee subjects were significantly different (p >0.05). The results of the questionnaire, presented in Fig. 9c, show an overall satisfaction of able-bodied subjects using the SH2-P and controlling different grips. A moderate level of fatigue was experienced by subjects at the end of the experiment (see Fig. 9c - Q5). These results are also supported by the feedback obtained from amputee subjects. Figure 10d shows the average score given for each question. The subjects gave an average score of *4.75 ±0.11* to Q1, Q2, Q3 and Q4. On average, also the level of fatigue experienced by the subjects was relatively low (*3.3 ±0.6*).

**Table 5:** Experimental Results of Amputee Subjects using SH2-P

		Subject 1	Subject 2	Subject 3
Box and Blocks Test		13	13	6
JTT	Writing	35.69	22.25	51.04
	Turn Cards	28.56	32.35	60
	Picking Small Obj	120	70.03	67.25
	Simulation Feeding	12.75	13.84	20.17
	Stacking Checkers	53.13	48.38	82.1
	Lift Light Can	14.12	16	28.44
	Lift Heavy Can	15.41	16.75	64
	Total	279.6	219.6	373
Questionaire		4.9	4.3	4.2

### Devices comparison

For able-bodied subjects, the average result of the BBT obtained with the SH2-P (*7.8 ±1.3*) is higher compared to the one with SH-P (*7.4 ±1.5*), but no statistically significant difference was found between the devices using a two-way analysis of variance with ANOVA (p >0.05). However, the outcome of the SH2-P is considerably lower compared to the score of the Delft Cylinder Hand (*26 ±8* after 15 trials) and the Ottobock Electric Hand (*25 ±3.5* for the slow device, *28 ±4* for the fast device). For the JTT, the results with the SH2-P (*321.2 ±33.7*) and the SH-P (*417.5 ±17.9*) were statistically different (p <0.05) and show about a 30% increase of the performance with the novel design.

The average results for subjects with limb loss show no significant difference between the SH2-P (*10.67 ±2.3*) and the SH-P (*9.6 ±1.4*) for the BBT. The score of the SH2-P is considerably lower compared to the results of [51], which analyze the performance of a body-powered Hosmer hook and a Motion Control Electric Device (respectively *49* and *20*), tested with a single subject with transhumeral amputation. However, the SH2-P shows higher score compared to [53], that presents the performance of a switch-controlled hand (*6*) and the Ottobock DMC Plus (*8* after 8 weeks training), and similar to the one obtained with the Michelangelo Hand [52] using direct control (*8.7 ±5.5* after home trial) and pattern recognition control (*11 ±6.1* after home trial).

The results of the JTT show about a 12% increase of the performance for the SH2-P compare to the single motor design (SH-P). As evident from the overview of the JTT subtask presented in Fig. 10c, the results obtained with the SH2-P overcome the one with SH-P especially in tasks which require a higher level of precision. In particular, a two-sampled t-test indicated that there is statistical difference (p <0.05) between the average score of the SH2-P (*85.76 ±10.5*) and the SH-P (*112.67 ±13.7*) in amputee subjects while performing the “grasp of small objects” JTT subtask. However, the SH-P was in average faster in the “lift of light/heavy cans” subtasks, but no statistical evidence was found (p >0.05). The outcomes of the SH2-P with the JJT are considerably better compared to the results of both devices presented in [53] (*621* with the switch-controlled hand, *567* with the Ottobock DMC Plus) and significantly different compared to the Michelangelo Hand [52], even after the home trial (*331 ±52.1* with DC, *332.3 ±69.3* with PR, p >0.05).

## Discussion

The performance reached with the SH2-P in subjects with and without limb loss are statistically comparable and demonstrated that a simple and soft hand design in combination with advanced pattern recognition could be a viable solution to balance system simplicity and dexterity. The introduction of a novel control modality, and especially with a limited training period, is undeniable challenging. Initially, subjects had to train how to switch directly between one muscle contraction to another but at the end of the training session, they were exploiting the advantages of this new strategy.

The results of the BBT show comparable performance between able-bodied and amputee subjects. The BBT was mostly used to evaluate the device usability more than hand dexterity, as generally the same grasp is repeated through the whole test. During both assessments, the subjects were free to use one gesture or a combination of them to complete the task, and the two groups of participants adopted different strategies to complete the BBT. While most of the able-bodied subjects completed the test using the same hand movement (power grasp), amputee subjects explored different configurations of the SH2-P with the continuous switching control method. Figure 8a shows an example. A subject used the index point configuration to move a block in a more convenient grasping position and then switched directly to power grip to grasp it, without the need to stop in a hand opening state. The wooden cube was then moved to the empty box and released, switching again directly from power grasp back to index point. These outcomes are also supported by the results of the JTT, that was selected to validate the hand functionality. All subjects successfully completed all the subtasks and, in average, amputee subjects performed better than able-bodied subjects. As presented in Fig. 8b, different grasping strategies were explored by participants and used to complete the JTT subtasks. Finally, the results of the questionnaire show very positive feedback from the users. They reported that they appreciated the adaptability to the object shape and the robustness of the device. Amputee subjects also noted features such as the device adaptability and dexterity, and they found the introduction of a continuous switching strategy to the conventional classifier promising. However, all the participants experienced a moderate level of fatigue during the experimental validation.

Moreover, the advanced dexterity of the SH2-P allows satisfactory performance also compared to the original design (SH-P). In the BBT, the average performance of the SH2-P are higher, but not statistically different from the one achieved with the 1 DoA design. This result is not surprising, since no need for switching DOF is required to complete this test. In the JTT, the advanced dexterity of the SH2-P allows to overcome the performance reached by amputee subjects using the SH-P. In particular, it is interesting to see how the extended capabilities of the SH2-P allow to get significantly better performance in tasks which requires more precision, as “grasp small objects” or “stacking checkers”, but worse performance in tasks as “lift of light/heavy cans”. The latter result could be a consequence of muscle fatigue or misclassified gesture predictions, and it is mostly influenced by the performance of subject 3 (as showed in Table 5). Generally, subjects 1 and 2 performed better than subject 3. This may be partially explained by the fact that the subject is a bodypowered user while the other two are myoelectric users. In general, the results were satisfactory enough to consider the system promising for future investigations, especially taking into account the limited training (less than 2 hours) and that the subjects were naive to the device and the control strategy. Indeed, it is also important to see that this work presents a first result of the SH2-P used by amputees and its evaluation through standardized assessment for prosthetic devices.

The analysis of the SH2-P in comparison with other studies from literature could give an additional interesting perspective. As presented in Tables 3 and 4, the outcomes of the SH2-P in the BBT are considerably lower compared to the Hosmer Hook [51] or the Delft Cylinder Hand [50], that allow higher grasping accuracy and proprioception thanks to the body-powered control. However, the BBT score obtained with the SH2-P and the continuous switching method is comparable to the outcomes found in other studies that use myoelectric control and state-of-the-art terminal devices with different levels of dexterity. While the BBT analyzes the device usability through a repeated action, the results of the JTT helps to highlight the function of the terminal device and control method for activities of daily living, that requires advanced dexterity. In this case, the proposed device and control method show a higher score compared to state-of-the-art myoelectrical controlled devices. In particular, the performance of the SH2-P are significantly different compare to the one of the Michelangelo Hand controlled using a conventional pattern recognition classifier, and this results could be possibly influenced by the use of the continuous switching method. Generally, the results on the SH2-P and the continuous switching method look promising for future extended investigations, even considering that the other studies used as a benchmark included longer training, several sessions or home trials.

**Fig. 11:**
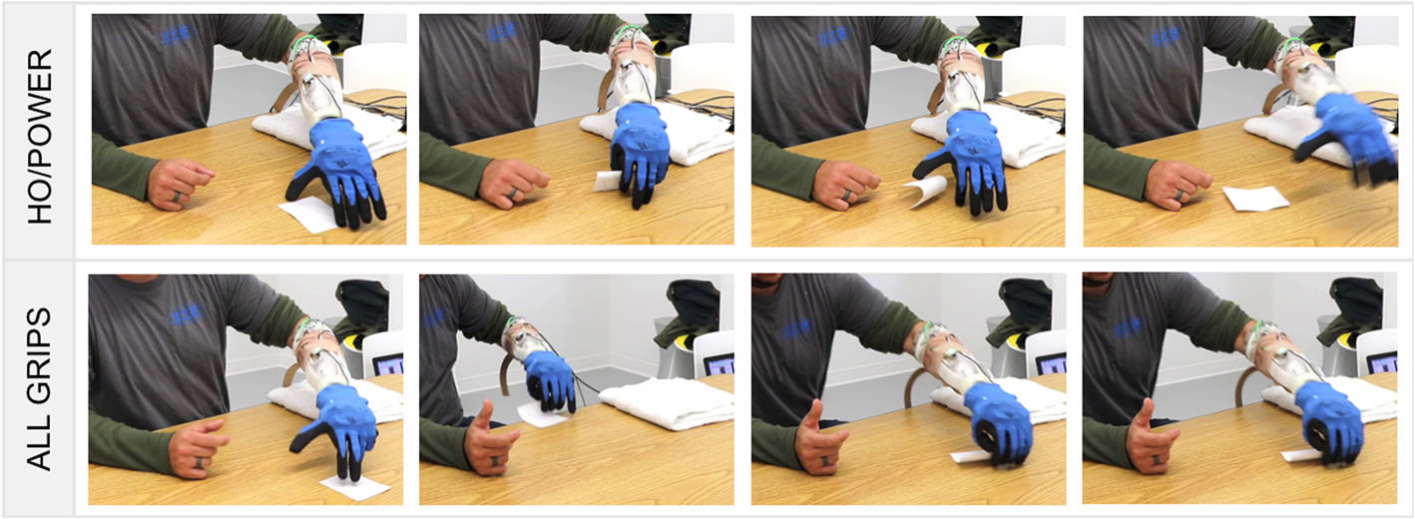
Amputee subject performing the “turn a card task” during different steps of the training session. In photo-sequence **a**-**d** the participant is intentionally using only power grasp, while **e**-**h** shows the result of combining index point and pinch grasp thanks to the continuous switching strategy. In the second case, the execution sequence is more natural

Despite valuable approaches that have been developed to investigate the possibility to combine the concept of postural synergies with different mapping strategies [26, 29], the proposed work evolves state-of-the-art in the use of a pattern recognition classifier as a method to extract intended commands. Moreover, the method is applied to a hand that physically implements multiple synergies, and does not reproduce them through the control of multiple motors connected to individual fingers. The use of prosthetic systems in real-world scenarios, which may experience harsh and irregular physical interactions with the environment, demands hardware which is physically resilient. The introduction of the augmented adaptive synergies directly in the mechatronics of the device has the potential to keep the robustness and device reliability experienced with the SH-P in a prototype which can perform an advanced level of dexterity but still maintaining the control intuitive and natural. This is visible in the example of Fig. 11 where, during the training session, the same task was performed with different gestures. In the photo sequence (a-d) the user was turning a card intentionally using only power grasp (as the SH-P can perform), while in the sequence (e-h) they were using the combination of all the hand gestures of the SH2-P. From this example, it is visible how, despite that the user can accomplish the task also using only a power grasp, with all the gesture and exploiting the continuous switching control the sequence is more natural. Please refer also to the video attachment for more details and examples of use.

The main limitation of this study is the lack of comparison of the continuous switching control method tested with other state-of-the-art advanced myoelectric prostheses and the same group of participants. While the comparison with the SH-P and other studies from literature helps to analyze the advantages and weakness of the proposed system, this study doesn’t show the performance of the continuous switching control method with other prosthetic devices. These aspects will be considered and evaluated in future investigations. Despite a small group of subjects that were involved in this study, the encouraging results allow us to confidently affirm that the method is certainly feasible and appreciated by the users. Future extensions of this works will include a larger group of participants and an advanced protocol, to examine the learning of subjects over multiple sessions and generalize our results.

## Conclusion

This paper explores the usability of a multi-synergistic soft prosthetic hand, the SoftHand 2 Pro, controlled using a pattern recognition classifier algorithm which implements a continuous switching control method. The combined system was validated using standard assessment in subjects with and without limb loss. Results show the potential of this approach also in comparison with the SoftHand Pro, which shares the same soft robotic technologies but implements only one single physical postural synergy. Indeed, if compared to the SoftHand Pro, the SoftHand 2 Pro shows improved fine grasp capabilities and better time performance, together with a useful set of different in-hand manipulation capabilities and gestures (e.g. index pointing). Moreover, the combination of state-of-the-art advanced controls with mechanical design simplifications demonstrated to be a promising approach to obtain a balance between control intuitiveness and device reliability, despite the presence of various novel manipulation skills. Finally, the proposed device and control method could show other benefits, as a possible reduction of compensatory motions. This more extensive evaluation will be part of future works, along with a higher number of participants.

## Data Availability

Not Applicable.

## Abbreviations

sEMG: Surface wlectromyographic
DoF: Degrees of freedom
TMR: Targeted Muscle Reinnervation
EMG: Electromyography
DoA: Degrees of actuation
SH-P: SoftHand Pro
SH2: SoftHand 2
SH2-P: SoftHand 2 Pro
CAPS: Control Algorithms for Prosthetics System
BBT: Box and Blocks Test
JTT: Jebsen-Taylor Hand Function Test
NA: not available

## Footnotes

1 CoApt LLC - https://www.coaptengineering.com/

2 Ottobock, Myo Plus Pattern Recognition - https://www.ottobock.com
